# Association between Work-Related Stress and Risk for Type 2 Diabetes: A Systematic Review and Meta-Analysis of Prospective Cohort Studies

**DOI:** 10.1371/journal.pone.0159978

**Published:** 2016-08-11

**Authors:** Hua Sui, Nijing Sun, Libin Zhan, Xiaoguang Lu, Tuo Chen, Xinyong Mao

**Affiliations:** 1 Institute of Basic Research of Integrative Medicine, Dalian Medical University, Dalian, Liaoning, P.R. China; 2 The Affiliated Hospital of Wuhan University of Science and Technology, Wuhan, P.R. China; 3 College of Basic Medicine, Nanjing University of Chinese Medicine, Nanjing, Jiangsu, P.R. China; 4 Department of Emergency Medicine, Zhongshan Hospital, Dalian University, Dalian, Liaoning, P.R. China; 5 Dalian Medical University, Dalian, Liaoning, P.R. China; University of Oxford, UNITED KINGDOM

## Abstract

**Background/Objective:**

The prevalence of type 2 diabetes is increasing rapidly around the world. Work-related stress is thought to be a major risk factor for type 2 diabetes; however, this association has not been widely studied, and the findings that have been reported are inconsistent. Therefore, we conducted a meta-analysis of prospective cohort studies to explore the association between work-related stress and risk for type 2 diabetes.

**Methods:**

A systematic literature search and manual search limited to articles published in English were performed to select the prospective cohort studies evaluated the association between work-related stress and risk for type 2 diabetes up to September 2014 from four electronic databases including PubMed, EMBASE, the Cochrane Library and Web of Science. A random-effects model was used to estimate the overall risk.

**Results:**

No significant association was found between work-related stress and risk for type 2 diabetes based on meta-analysis of seven prospective cohort studies involving 214,086 participants and 5,511 cases (job demands: relative risk 0.94 [95% confidence interval 0.72–1.23]; decision latitude: relative risk 1.16 [0.85–1.58]; job strain: relative risk 1.12 [.0.95–1.32]). However, an association between work-related stress and risk for type 2 diabetes was observed in women (job strain: relative risk 1.22 [1.01–1.46]) (*P* = 0.04). A sensitivity analysis conducted by excluding one study in each turn yielded similar results. No publication bias was detected with a funnel plot despite the limited number of studies included in the analysis.

**Conclusions:**

The results of this meta-analysis did not confirm a direct association between work-related stress and risk for type 2 diabetes. In subgroup analyses we found job strain was a risk factor for type 2 diabetes in women.

## Introduction

Type 2 diabetes is one of the most prevalent chronic diseases, ever-increasing worldwide, especially in developing countries. It is one of the major public health challenge of the 21st century. The 7th edition of the Diabetes Atlas, released by the International Diabetes Federation on World Diabetes Day, reports that the number of worldwide diabetes cases reached 415 million in 2015, and is expected to rise to 642 million by 2040. Researchers predict that annual health care expenditure may increase from the current figure of $673 billion to $802 billion by 2040 [1]. As early as the 17th century, Thomas Willis, the famous English physician, linked diabetes to emotional factors such as prolonged sorrow [2], which is now known as psychosocial stress. The American psychiatrist Dr. W. Menninger first tested Willis’s hypothesis and mentioned the existence of psychogenic diabetes in 1935 [3]. Almost 40 years later, psychosocial stress was attracting growing attention, and the famous psychiatrist George Engel noted the shift from a traditional biomedical to a biopsychosocial model [4].

Researchers have confirmed numerous risk factors for type 2 diabetes, including family history, low exercise levels [5,6], increased weight [7], heavy smoking [8] and alcohol consumption [9]. The etiology of type 2 diabetes is complex, and work-related stress may contribute to, or increase, the risk of its development. Some evidences have proved that psychosocial stress at work is an important risk factor for heart disease [10–13]. However, the results of the studies examining the association between work-related stress are inconsistent. Previous meta-analyses did not support the hypothesis that work-related stress increases the risk for type 2 diabetes [14,15].

The job strain model, proposed by Karasek and Theorell, provides a theoretical model for assessing the role of work stress in the onset of type 2 diabetes. The job strain model includes job demands (the summation of psychosocial work stressors), decision latitude/job control (the individual’s decision authority at work), and job strain (the combination of high levels of job demands and low levels of job control) [16].

Therefore, we performed a systematic review and meta-analysis of prospective cohort studies for the following purposes: 1) to review recent evidence on the relationship between work-related stress and incidence of type 2 diabetes; and 2) to examine whether this relationship differs according to the different kinds of work-related psychosocial factors and participant characteristics.

## Materials and Methods

### Data sources and searches

To identify observational epidemiological studies that investigated the association between work-related psychosocial stress and the incidence of type 2 diabetes, we followed guidelines for meta-analysis of observational studies in epidemiology [17]. Two investigators independently conducted a systematic literature search of English language articles published before September 2014 using the following bibliographic databases and time ranges: PubMed from 1948, EMBASE from 1974, the Cochrane Library from 1993, and Web of Science from 1900.

The main search strategy included medical subject heading terms or text words for “outcome” (diabetes mellitus, type 2 OR diabetes mellitus) and “exposure” (work stress OR occupational stress OR work-related psychosocial stress OR work-related psychosocial factors OR psychosocial work environment OR work characteristics OR work demand OR decision latitude OR job control OR job strain OR related terms) and “design type” (prospective studies OR cohort studies OR longitudinal studies OR incidence or follow-up studies). Furthermore, we examined the references of retrieved original articles and reviews to identify additional relevant studies. When necessary, we attempted to contact the authors for additional information. The supporting PRISMA checklist is available as supporting information (see S1 Checklist).

### Study selection

The current systematic review and meta-analysis was restricted to prospective studies because case-control and cross-sectional studies are known to be subject to selection and recall bias, and cannot definitively determine the association between exposure and outcome. Published articles were included if they met the following criteria: (1) used a prospective cohort study design; (2) assessed the incidence of type 2 diabetes; (3) presented a measure of work-related stress (including job demands, decision latitude/job control, job strain); and (4) reported the odds ratios (OR) or relative risk (RR), or hazard ratio (HR) and its 95% confidence interval (CI), for highest *versus* lowest/non-level of work-related psychosocial stress. Studies were excluded for the following reasons: (1) the outcome measure was prediabetic state or metabolic syndrome rather than type 2 diabetes; (2) the study focused on control as opposed to incidence of type 2 diabetes; (3) or exposure was related to working hours or overtime work, night work or shift work, trauma, burnout, violence or accidents at work, social network outside the workplace, psychological distress such as anxiety and depression, personality, or coping style. If publications were duplicated, or if the same cohort was analyzed in more than one article, the study with the longer follow-up period, higher quality and more detailed information for both exposure and outcome was included.

### Data extraction and quality assessment

We used a standard data extraction form to collect the following information: first author’s name, publication year, cohort designation, country, follow-up duration (years), participants’ sex and age, number of participants and cases, evaluation of exposure and outcome, most fully adjusted risk estimates with corresponding 95% CIs for the highest *versus* the lowest category of type 2 diabetes in relation to work-related stress category and sex, and the main confounding factors controlled for in the analysis. Study selection and data extraction were conducted by two experienced investigators independently. Any differences were resolved by consensus.

The Newcastle-Ottawa Quality Assessment Scale for cohort studies[18], in which a study is judged mainly according to the selection of study groups, comparability of the groups, and ascertainment of the exposure and outcome, was used to assess the quality of observational studies. The highest score possible is nine stars, we considered a study with six stars or above as a high-quality study.

### Statistical Analysis

The RRs were used to examine associations across studies. To accomplish this, the HRs were regarded directly as RRs. The ORs were transformed into RRs using the following formula:

$$RR=OR/\left[\left(1-P_{0}\right)+\left(P_{0}\times OR\right)\right]$$

Where P_o_ is the incidence of the outcome of interest in the unexposed group [19]. We found that outcomes across studies were relatively uncommon as the ORs mathematically approximated the RRs; therefore, we considered ORs as surrogates for RRs [20]. Statistical heterogeneity across studies was examined by Cochran’s Q statistics and quantified by *I*^*2*^ statistics. For the Q statistic, a *P* value < 0.10 was considered to indicate statistically significant heterogeneity. The *I*^*2*^ values vary from 0 to 100, with a level of 25% or less considered low heterogeneity, about 50% medium heterogeneity, and about 75% or greater high heterogeneity [21]. The fixed-effect model (inverse-variance method) [22] was used to compute summary RRs across studies. When statistically significant heterogeneity was detected, the pooled risk estimate was based on the random-effects model [23] because both within and between variations were present across the studies.

Meta-analyses generally include only one effect size per study. If more than one kind of work-related psychosocial factor was analyzed in a single article, we treated these factors as subjects of separate studies. Results that were presented separately by sex were entered into the meta-analyses as independent studies. To obtain more robust summary results, we calculated the pooled risk estimates and 95% CI of type 2 diabetes incidence for job demands, decision latitude, and job strain separately for highest *versus* lowest category [24] of work-related psychosocial factors.

Subgroup analyses were carried out to evaluate possible sources of statistical heterogeneity and to examine the potential impacts of participant characteristics (sex) on the relationship between work-related stress and incidence of type 2 diabetes. To assess the robustness of this relationship, we also conducted sensitivity analyses in which single studies were sequentially omitted and the remaining studies were synthesized to evaluate the influence of individual studies on the summary risk estimate.

Visual inspection of a funnel plot was used to detect publication or other types of bias. In the presence of publication bias, the funnel plot should be asymmetrical [25]. Meta-analysis was performed using Review Manager version 5.2 (The Nordic Cochrane Centre, The Cochrane Collaboration, Copenhagen, Denmark). All statistical tests were two-sided and *P*<0.05 was considered statistically significant.

## Results

### Literature search

**Fig 1:**
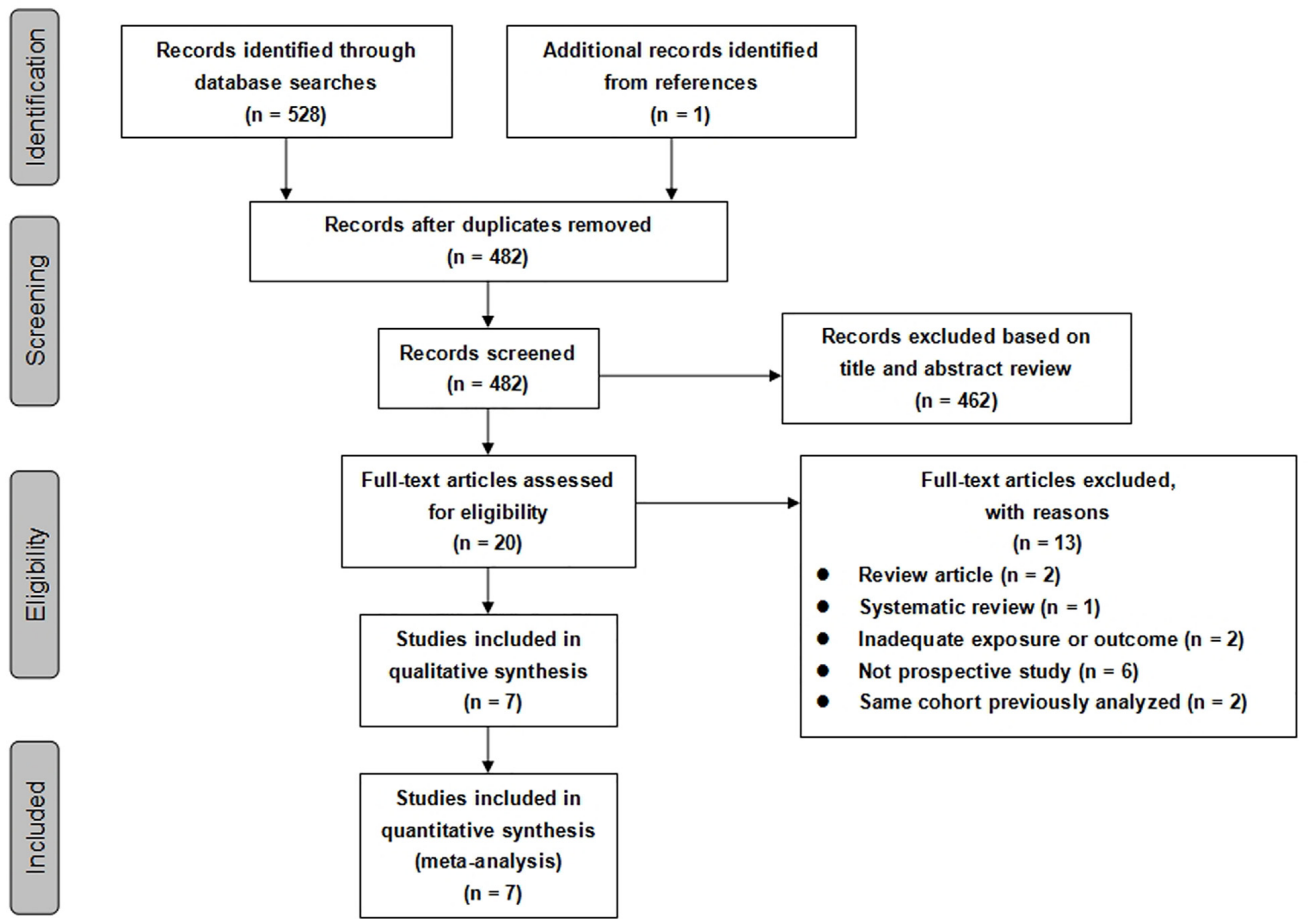
Flow chart for selection of articles included in the meta-analysis.

A flow diagram is shown in Fig 1. The literature search identified a total of 528 articles: 51 from PubMed, 40 from EMBASE, 0 from the Cochrane Library and 437 from Web of Science. Manual searching of the references yielded 1 additional record. A total of 47 duplicate articles were excluded, leaving 482 articles for screening. Among these, 462 articles were removed at the first screening on the basis of their titles or abstracts, leaving 20 studies for full-text review. In this review, 13 studies were excluded for various reasons (review, systematic review, inadequate exposures or outcomes, not prospective cohort studies, or data from the same cohort). There were seven prospective cohort studies [26–32] were included in this systematic review and meta-analysis. Additionally, three articles [28,33,34] were based on the same cohort as the Whitehall II study, but with different durations of follow-up. To avoid duplicate inclusion of study population, we selected the studies with a longer follow-up and more complete analysis [28].

### Study characteristics

We identified seven prospective cohort studies involving 214,086 participants and 5,511 cases. Type 2 diabetes was reported as the main outcome in these articles, except for one study [29] that did not identify diabetes type (given the high prevalence of type 2 diabetes, in particular among older cohorts, this restriction was unlikely to impact the results [35]).The characteristics of the studies included in our analysis are presented in Table 1. The selected studies were published between 1999 and 2014. Regarding study location, four were performed in Europe [28,30–32], one in the United States [27], one in Canada [29], and one in Japan [26]. Four studies surveyed population-based cohorts [29–32] and three surveyed occupational cohorts [23–25]. The average follow-up duration ranged from 5.8 to 10.3 years. The number of subjects varied from 2,597 to 124,808, and the number of cases investigated in the studies ranged from 34 to 5,511. Five studies recruited both men and women [28–32], one consisted of men only [26], and one consisted of women only [27]. Mean age at baseline ranged from 18 to 66 years. Overall, six reported results on the relationship between job demands and type 2 diabetes [28–30], six on decision latitude [28–30], and eight on job strain [26–28,30–32]. All studies used the Karasek Job Content Questionnaire to assess work-related psychosocial factors. Although type 2 diabetes ascertainment was primarily based on self-reports from physician diagnoses, most of the cases were comfirmed in medical records. Most studies were adjusted for a wide range of confounders, including age, body mass index (BMI), education, physical activity, smoking, alcohol consumption and family history of diabetes. Additionally, the study quality score ranged from six to eight.

**Table 1:** Main characteristics of cohort studies included in the meta-analysis.

Study	Country/Cohort Designation	Follow-up (y)	Participants (cases)	Age (y)	Psychosocial work characteristics	Adjusted RR (95%CI)	Exposure assessment	Case ascertainment	Adjustment for potential confounders	Quality score
Kawakami, 1999 [26]	Japan/Electrical Co.	8	2597 men (34)	18–60	Job strain	M:1.34 (0.50,3.55)	JCQ	OGTT, FPG	Age, education, BMI, alcohol consumption, smoking, leisure time physical activity, family history	8
Kroenke 2007 [27]	USA/NHS II	5.8	62574 women (365)	29–46	Job strain	F:1.11 (0.80,1.52)	JCQ	Confirmed self-report	Age, BMI, family history of diabetes, work hours, rotating night-shift work, hours at work sitting, job support, hours per week of, work at home, leisure-time physical activity, smoking, alcohol intake, trans-unsaturated fat intake, glycemic load, caffeine intake, marital status, number of children, menopausal status, vitamin supplementation, aspirin use	6
Heraclides 2009 [28]	UK/Whitehall II	11.6	4166 men and 1729 women (308)	35–55	Job demands, Decision latitude, Job strain	Job demands: T: 0.88(0.70–1.10) M: 0.82 (0.63–1.07) F: 1.06 (0.70–1.62) Decision latitude: T: 0.94(0.75–1.18) M: 0.86 (0.66–1.13) F: 1.09 (0.70–1.69) Job strain: T: 1.04 (0.80–1.34) M: 0.82 (0.59–1.15) F: 1.59 (1.03–2.45)	JCQ	Confirmed self-report, OGTT, use of diabetes medication	Age	7
Smith, 2012 [29]	Canada/CHS	9	3691 men and 3752 women (639)	35–60	Job demands, Decision latitude	Job demands: T:–M: 1.30 (0.81–2.08) F: 1.32 (0.75–2.33) Decision latitude: T:–M: 0.92 (0.56–1.51) F: 2.04 (1.15–3.61)	JCQ	Physician reported	Age, immigration status, ethnicity, marital status, urban or rural living location, education, heart disease at baseline, hypertension at baseline, depression at baseline, activity limitations at work due to health problems, shift schedule, weeks worked, multiple jobs, physical activity at work, smoking, alcohol, leisure time physical activity, fruit and vegetable consumption, BMI	8
Eriksson, 2013 [30]	Sweden/SDPP	8–10	2227 men and 3205 women (171)	35–56	Job demands, Decision latitude, Job strain	Job demands: T:0.70 (0.50–1.10) M: 0.50 (0.30–0.90) F: 1.00 (0.50–2.00) Decision latitude: T: 1.30 (0.80–2.10) M: 0.90 (0.50–1.70) F: 2.40 (1.10–5.20) Job strain: T: 0.80 (0.50–1.40) M: 0.50 (0.30–0.90) F: 2.10 (0.90–4.80)	Swedish version of the JCQ	OGTT	Age, Educational level, psychological distress, family history of diabetes, BMI, physical activity, smoking, civil status	8
Huth, 2014 [31]	Germany/ MONICA	12.7	3350 men and 1987 women (291)	29–66	Job strain	Job strain: T: 1.45 (1.00–2.10) M:–F: –	JCQ	Self-report	Age, sex, baseline survey, BMI, low education, physical intensity of work, parental history of diabetes, physical inactivity, smoking, alcohol intake, living alone	8
Nyberg, 2014[32]	Finland, France, Denmark, Sweden, UK/ IPD-Work	10.3	54006 men and 70802women (3703)	35–53	Job strain	Job strain: T: 1.15 (1.06–1.25) M: 1.19 (1.06–1.34) F: 1.13 (1.00–1.28)	JCQ, DCQ	OGTT, hospital records, mortality register, medication reimbursement, self-report	Age, sex, SES, working hours, BMI, leisure-time physical activity, smoking, alcohol consumption	8

NHS II, Nurses Health Study; CHS, Community Health Survey; SDPP, Stockholm Diabetes Prevention Program; MONICA, Monitoring Trends and Determinants of Cardiovascular Disease; IPD-Work, Individual-Participant-Data meta-analysis of Work; JCQ, Job Content Questionnaire; DCQ, demand control questionnaire; OGTT, oral glucose tolerance test; FPG, fasting plasma glucose; BMI, body mass index; SES, socioeconomic status.

### Main analysis

**Fig 2:**
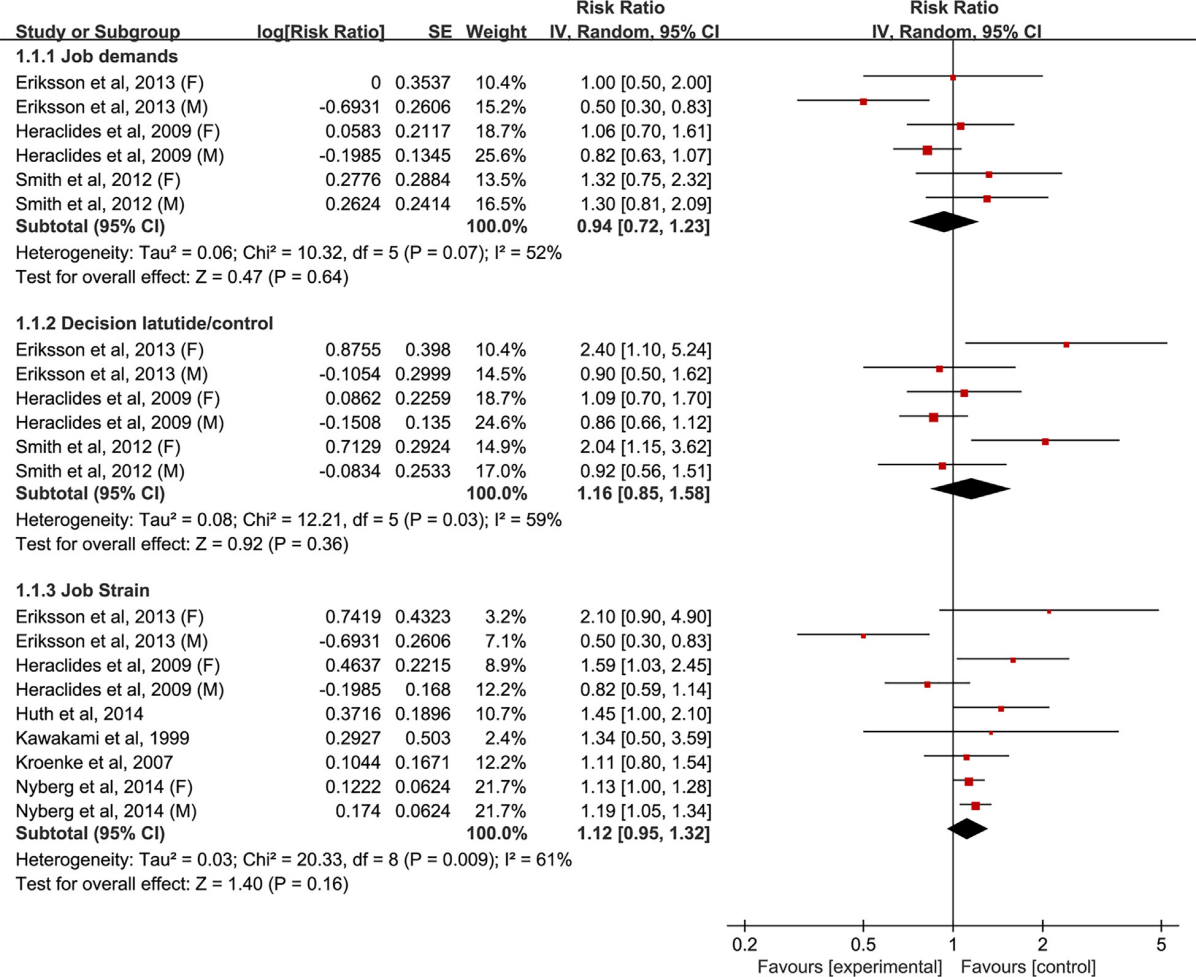
Forest plot of the association between the job strain model and type 2 diabetes risk. SE, standard error; CI, confidence interval; M, male; F, female.

**Fig 3:**
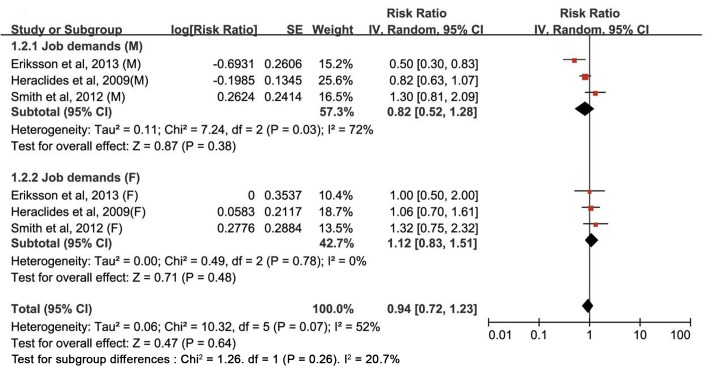
Estimates of type 2 diabetes associated with job demands in men and women. SE, standard error; CI, confidence interval; M, male; F, female.

**Fig 4:**
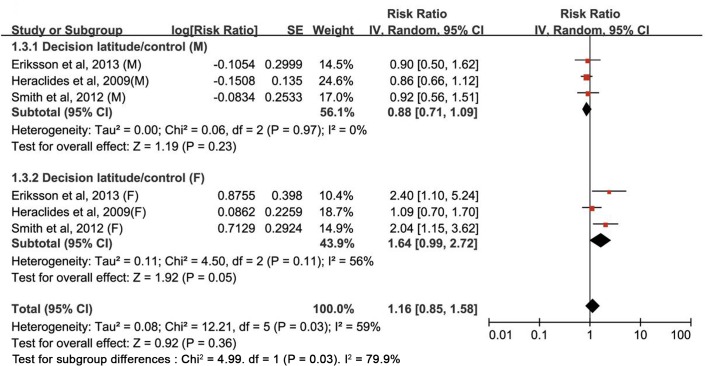
Estimates of type 2 diabetes associated with decision latitude/control in men and women. SE, standard error; CI, confidence interval; M, male; F, female.

**Fig 5:**
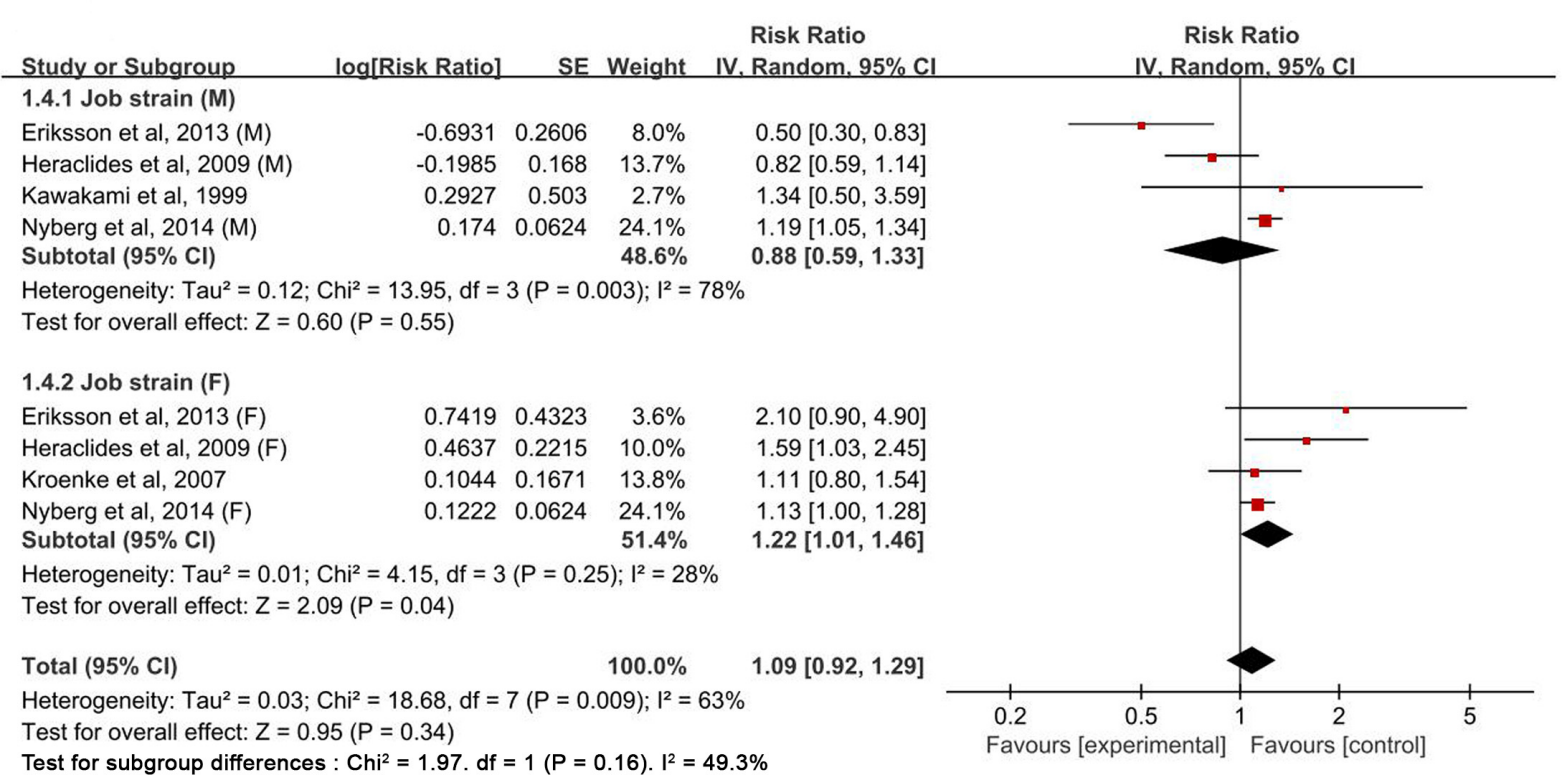
Estimates of type 2 diabetes associated with job strain in men and women. SE, standard error; CI, confidence interval; M, male; F, female.

The multivariable-adjusted RRs of type 2 diabetes for each study and all studies combined for the highest *versus* the lowest category of four components of the job strain model are shown in Figs 2–5. The results of subgroup analyses stratified by sex are shown in Table 2.

**Table 2:** Subgroup analyses relating work stress to type 2 diabetes by sex.

Type of work stress	Number of studies	RR	(95%CI)	*P*	*P*_*heterogeneity*_	*I*^*2*^(%)	*P*_interaction_	References
**Job demands**							0.26	
Total	6	0.94	(0.72–1.23)	0.64	0.07	52.0		28–30
Male	3	0.82	(0.52–1.28)	0.38	0.03	72.0		28–30
Female	3	1.12	(0.83–1.51)	0.48	0.78	0		28–30
**Decision latitude/control**							0.03	
Total	6	1.16	(0.85–1.58)	0.36	0.03	59.0		28–30
Male	3	0.88	(0.71–1.09)	0.23	0.97	0		28–30
Female	3	1.64	(0.99–2.72)	0.05	0.11	56.0		28–30
**Job strain**							0.16	
Total	8	1.12	(0.95–1.32)	0.16	0.0009	61.0		26–28, 30, 31, 32
Male	4	0.88	(0.59–1.33)	0.55	0.003	71.0		26, 28, 30, 32
Female	4	1.22	(1.01–1.46)	0.04	0.25	28.0		27, 28, 30, 32

RR, relative risk; CI, confidence interval.

#### Job demands

In a pooled analysis of six studies, no significant relationship was observed between job demands and type 2 diabetes risk (RR: 0.94 [95% CI: 0.72–1.23]) (Fig 2). In subgroup analyses by sex, no significant association was in neither men (RR: 0.82 [0.52–1.28]) nor women (RR: 1.12 [0.83–1.51]). Substantial statistical heterogeneity was found among the studies (*P*_heterogeneity_ = 0.07, *I*^*2*^ = 52.0%); this heterogeneity remained significant for men (*P*_heterogeneity_ = 0.03, *I*^*2*^ = 72.0%), but not for women (*P*_heterogeneity_ = 0.78, *I*^*2*^ = 0.0%) (Fig 3 and Table 2). Sensitivity analyses evaluating the robustness of the association by sequentially omitting each study yielded a narrow range of RRs, from 0.93 (0.69–1.27) to 0.98 (0.82–1.18).

#### Decision latitude

The pooled results based on the six studies showed that the estimate of the association between decision latitude and the risk of type 2 diabetes was not significant (RR: 1.16 [0.85–1.58]) (Fig 2). In subgroup analyses by sex, no association was found between low decision latitude and high risk of type 2 diabetes both in women (RR: 1.64 [0.99–2.72]) (*P* = 0.05) and men (RR: 0.88 [0.71–1.09]) (*P* = 0.23). Statistically significant heterogeneity was observed across studies (*P*_heterogeneity_ = 0.03, *I*^*2*^ = 59.0%); this heterogeneity remained substantial for women (*P*_heterogeneity_ = 0.11, *I*^*2*^ = 56.0%), but not for men (*P*_heterogeneity_ = 0.97, *I*^*2*^ = 0.0%) (Fig 4 and Table 2). Sensitivity analyses assessing the robustness of the association by sequentially omitting each study yielded a narrow range of RRs, from 0.97 (0.80–1.17) to 1.05 (0.80–1.38).

#### Job strain

In a pooled analysis of nine studies, no association was found between job strain and risk of type 2 diabetes (RR: 1.12 [0.95–1.32]) (Fig 2). When stratified by sex, the studies for women showed job strain is the risk of type 2 diabetes (RR: 1.22 [1.01–1.46]) (*P* = 0.04), but not for men (RR: 0.88 [0.59–1.33]). Significant heterogeneity was evident among studies (*P*_heterogeneity_ = 0.0009, *I*^*2*^ = 61.0%); this heterogeneity showed a reduction for women (*P*_heterogeneity_ = 0.25, *I*^*2*^ = 28.0%), but remained significant for men (*P*_heterogeneity_ = 0.003, *I*^*2*^ = 78.0%) (Fig 5 and Table 2). Sensitivity analyses testing the robustness of our findings by sequentially excluding each study yielded a narrow range of RRs, from1.08(0.92–1.28)to 1.17(1.05–1.32)

### Publication bias

Although the number of studies included in the analysis was limited, no publication bias was detected using a funnel plot.

## Discussion

### Main findings

Consistent with previous meta-analyses [14], our results did not confirm the association between work-related stress and risk for type 2 diabetes. We included prospective cohort studies to minimize the possibility of selection and recall biases. Furthermore, we conducted stratified analyses to explore sources of heterogeneity which improved the clinical value of this research [36]. More importantly, the association observed in women implied that sex may serve as an effect modifer of the association between work-related stress and type 2 diabetes. In our subgroup analyses, the effect of sex on the association between job strain and type 2 diabetes was obvious(P = 0.04). So job strain was a risk factor for type 2 diabetes in women.

At first we did not include the paper published by Nyberg et al. [32]. The results showed work-related stress was not a risk for type 2 diabetes (job demands: RR 0.94 [95% confidence interval 0.72–1.23]; decision latitude: RR 1.16 [0.85–1.58]; job strain: RR 1.11 [0.81–1.53]). When stratified by sex, the studies for women showed no effect of job strain on the development of type 2 diabetes (RR: 1.38 [0.99–1.91]) (P = 0.06). This study[32] is a multi center study, the researchers extracted individual-level data for 124,808 diabetes-free adults from 13 European cohort studies participating in the IPD-Work Consortium. Later, we included this article. Although the hypothesis that work-related psychosocial stress increases the risk for type 2 diabetes cannot be supported, in the subgroup analyses we found job strain was a risk factor for type 2 diabetes in women, (RR: 1.22 [1.01–1.46]) (P = 0.04).

Significant statistical heterogeneity was detected among these studies, further sensitivity analysis yielded similar results across studies. It was not surprising that significant heterogeneity was found due to the variations in ethnicity, method of case ascertainment, the components of the job strain model compared, and adjustment among studies. In the subgroup analyses by sex, heterogeneity showed a reduction. This suggests that sex may, at least partially, be responsible for heterogeneity.

### Possible mechanism

A causal relationship between work-related stress and the incidence of type 2 diabetes is biologically credible. Type 2 diabetes is associated with work-related psychosocial stress via two key patterns including dysregulation of neuroendocrine and lifestyle-related factors. Dysregulation of neuroendocrine pathways maybe the most important mechanism. Work-related psychosocial stress may increase the risk for type 2 diabetes through chronic activation of the hypothalamus–pituitary–adrenal axis and the sympathetic nervous system, lead to the release of sympathetic hormones and glucocorticoids such as cortisol, resulting in increased hepatic glucose output, decreased insulin secretion, insulin resistance and visceral obesity. Chronic psychosocial work-related stress is associated with elevated cortisol levels [37]. Cortisol can influence the regulation of blood glucose by altering the body’s release of insulin and sensitivity to insulin, resulting in an increased risk of type 2 diabetes [38]. The indirect mechanism is the changes of lifestyle-related factors, such as poor eating behavior, physical inactivity, smoking and alcohol consumption [39–41], all of which have been shown to increase the risk for type 2 diabetes. However, the results regarding the association between work stress and type 2 diabetes are inconsistent. For example, one previous meta-analysis could not support the hypothesis that work-related stress increased the risk for type 2 diabetes [14]. Findings from a large pan-European dataset suggested that job strain is a risk factor for type 2 diabetes in men and women, independent of lifestyle-related factors [31]. Furthermore, long hours of overtime work may not be associated with increased prevalence of diabetes among Japanese [42].

In subgroup analyses we found job strain was related to risk of type 2 diabetes in female. Some previous researches have similar results [15,27,30]. Eriksson and colleagues [30] speculated that sex-based differences regarding the effects of work-related stress could be attributable to a divergence in gender roles outside work, as women tend to spend more time than men on household responsibilities and childcare, thereby not having the same opportunity to relax [43–47]. Additionally, some evidence suggest that women may be more prone to the health impact of chronic psychosocial stress due to gender-specific psychoneuroendocrine activation [43,48–50]. Among a subsample from the Whitehall II study, men and women had similar salivary cortisol levels during weekends, but women had significantly higher cortisol levels than men during working days [51]. Similar results have been observed in one Italian [52] and one German [53] study, which are regarding gender-specific cortisol responses to chronic work-related stress. The generation of job stress has a different pattern in men and women. Both quantitative and qualitative (intellectual and emotional) demands determine occupational stress in women, while only quantitative demands are stressors for men[54]. Support has a significantly stronger impact on levels of job stress in the workplace among women[15, 54].

Although we are unable to confirm that work-related stress increases the risk for type 2 diabetes, women in high demand and low-control occupations could be at a greater risk of negative health consequences [55].

### Limitations

Several limitations should be acknowledged. First, observational studies cannot establish causal relationships between exposure factors and outcome events. Second, residual confounding remains a concern. A wide range of potential confounders were adjusted for in most studies, including age, BMI, physical activity, smoking, alcohol consumption and family history of diabetes. However, stress indices unrelated to work (such as depression, anxiety, life events, stress-prone personality, coping style and sleeping problems) and factors in other work-related stress models (such as work hours, rotating night-shift work, effort-reward imbalance and job insecurity) were not sufficiently adjusted. Therefore, we could not fully exclude the possibility that unadjusted confounders had effects on the association with risk for type 2 diabetes. Third, misclassification bias may have attenuated the association. Because work-related stress assessment was based on self-administered questionnaires, misclassification of work-related stress was inevitable. Case ascertainment in most studies was based on self-reports, so there could have been misclassification of type 2 diabetes cases. Additionally, one study [29] that did not distinguish between different types of diabetes might have weakened the relationships.We limited studies to publications in English. Multiple stress indicators across studies may have increased the risk of selective publication. Publication bias was thus conceivable, even though it was not detected using a funnel plot [56]. In addition, there are some other limitations. Sample sizes ranged from 2597 to 124808, thus the largest study [32] may have largely determined the results. A wide age range (18 to 66 years) and a wide range of follow-up time (5.8 to 12.7 years) limit the research. There is also only one Asian sample from Japan[26], limiting the study relevance for Asian populations.

### Clinical implications

Until now, large scale and well-conducted randomized controlled trials, the gold standard for investigating a causal association [57], have not been conducted to directly assess the effect of work-related stress on risk for type 2 diabetes. With mounting observational studies of the diabetogenic effect of work-related stress, randomized controlled trials should focus on the reduction of work-related stress, either by improving the work environment or by improving individual coping strategies, especially for women [15].

In conclusion, the hypothesis that work-related psychosocial stress increases the risk for type 2 diabetes could not be supported from the meta-analysis. In a subgroup analysis we found job strain to increase the risk of type 2 diabetes in women. Although the more general hypothesis was not supported, our results suggest that it may depend on a number of factors, and thus further study is needed.

## Supporting Information

S1 Checklist PRISMA Checklist. (DOC)
